# Respiratory Syncytial Virus in Pregnancy: An Obstetrics View

**DOI:** 10.3390/pediatric16040078

**Published:** 2024-10-17

**Authors:** Mattia Dominoni, Barbara Gardella, Arsenio Spinillo

**Affiliations:** 1 Department of Clinical, Surgical, Diagnostic and Paediatric Sciences, University of Pavia, 27100 Pavia, Italy; b.gardella@smatteo.pv.it (B.G.); spinillo@smatteo.pv.it (A.S.); 2 Department of Obstetrics and Gynecology, Fondazione IRCCS Policlinico San Matteo, 27100 Pavia, Italy

Respiratory syncytial virus (RSV) represents one of the most prevalent causes of lower respiratory tract infection in newborns and children by the time they are two years old, with a peak rate of hospitalization in those between two and three months of age and a high risk of morbidity and mortality, especially under the age of six months of life [1,2,3]. Infants who are premature, immunocompromised, or who have congenital heart disease, congenital lung disease, or congenital airway defects are most vulnerable to severe RSV-related conditions [4,5,6]. Compared to the general population, the incidence of RSV diseases and severe infection is higher in infants that are non-Hispanic Black, American Indian, or Alaska Native [7]. In addition, data from the literature have shown that childhood RSV exposure increases the risk of long-term respiratory morbidity, such as allergies, asthma, and impaired respiratory function, with a decrease in quality of life and an increase in the consumption of medical treatment [8].

Currently, the absence of effective methods to lower RSV-related morbidity and mortality has led to renewed interest on the development of RSV vaccination. Up until recently, the only method of preventing RSV in infants was to administer monthly injections of palivizumab (a humanized monoclonal antibody), motavizumab (a higher-potency monoclonal antibody), or nirsevimab (a long-acting monoclonal antibody) which has been recently approved specifically to prevent severe RSV disease in infants under 8 months old, born during or about to begin their first RSV season, and in children 8–19 months old who are at risk of developing severe RSV disease and starting their second RSV seasons. All these antibodies can bind to and neutralize the RSV fusion protein in high-risk infants in order to provide passive immunity [9,10,11,12].

In pregnancy, the rationale behind RSV vaccination is due to the characteristics of respiratory syndrome, which occurs in the first 2–3 months of life, prior to vaccine administration for newborns. Administering the vaccine to the mother protects the infant through trans-placental immunization. In the other words, the first line of defense against RSV infection in infants is maternal RSV-specific immunoglobulin G (IgG) antibodies, which are transferred to the fetus through the Fc receptors of syncytiotrophoblast cells in the chorionic villi [13,14]. Although maternally generated RSV-specific IgG antibodies in a newborn have a very short half-life (28–40 days), the high concentration of maternal RSV antibodies still allows immunity in newborns against severe RSV-related conditions [15,16].

In recent years, the development of vaccinations has been focused on the RSV fusion protein because it produces a stronger immunogenic response and larger levels of neutralizing antibody titers. In 2020, a phase III, randomized, placebo-controlled clinical study using the intramuscular injection of RSV fusion protein nanoparticles assessed the effectiveness of RSV immunization in pregnancy. Healthy women with low-risk singleton pregnancies and gestational ages between 28 and 36 weeks prior to the seasonal circulation of RSV were enrolled in the trial. The study reported that, in the first 90 days of life, serious lower respiratory tract infections had a prevalence of 1.5% in the vaccine group and 2.4% in the control group (vaccine effectiveness, 39.4%; 97.52% CI, −1.0 to 63.7; 95% CI, 5.3 to 61.2). RSV-related lower respiratory tract infection was associated with severe hypoxemia in 0.5% and 1.0% of cases (vaccine efficacy, 48.3%; 95% CI, −8.2 to 75.3). Hospitalization rates for RSV-related lower respiratory tract infection were 2.1% and 3.7%, respectively (vaccine efficacy, 44.4%; 95% CI, 19.6 to 61.5). In this way, RSV F protein nanoparticle vaccination in pregnant women did not fulfill the predetermined success criterion for efficacy against RSV-associated, medically relevant lower respiratory tract illness in infants in the first 90 days [17].

In 2023, a phase III, randomized, double-blind trial evaluated a single intramuscular injection of 120 μg of a bivalent RSV perfusion F protein-based (RSVpreF) vaccine vs. a placebo in pregnant women at 24 through 36 weeks’ gestation in order to evaluate the efficacy of vaccination in reducing severe and not-severe RSV-associated lower respiratory disease in infants within 90, 120, 150, and 180 days after birth. According to the trial, the RSVpreF vaccination delivered during pregnancy effectively prevented severe RSV-associated lower respiratory tract infection in babies, with no safety issues observed. The level of vaccine effectiveness was 81.8% (99.5% CI, 40.6–96.3) against severe RSV-associated lower respiratory tract disease in infants within 90 days of birth and 69.4% (97.58% CI, 44.3–84.1) within 180 days. The vaccine efficacy level for not-severe RSV-associated lower respiratory tract disease in the first 90 days of life was 57.1% (99.5% CI, 14.7–79.8), falling short of the pre-specified success criterion. The immunization group had a not statistically significant higher risk of pre-term birth of 5.7% (95% CI, 4.9–6.5), compared to the control group’s risk level of 4.2% (95% CI, 3.3–5.3) [18].

For this reason, in September 2023, the FDA approved Abrysvo (respiratory syncytial virus vaccination) for pregnant individuals to prevent severe and not-severe lower respiratory tract disease caused by respiratory syncytial virus (RSV) in infants from birth to 6 months of age in a single dose [19]. This came after nirsevimab, which was approved for use in newborns in July of 2023 [11]. The American College of Obstetricians and Gynaecologists and the Society for Maternal-Fetal Medicine have both endorsed this suggestion [12].

In 2024, a randomized, phase III trial evaluated the efficacy and safety of RSV pre-fusion F protein-based maternal vaccination (RSVPreF3-Mat) between 24 weeks 0 days and 34 weeks 0 days of gestation. The primary outcomes included severe or not-severe RSV-associated lower respiratory tract disease in infants from birth to 6 months of age, as well as safety in infants from birth to 12 months of age. After the insurgence of a greater risk of pre-term delivery in the vaccine group, enrolment and immunization were halted early. The author attested that the risks of severe and not-severe RSV-associated lower respiratory tract disease among infants were lower with the vaccine but that the risk of pre-term birth was higher in the vaccine group. The vaccine efficacy level was 65.5% (CI 95%, 37.5–82.0) for any RSV-associated lower respiratory tract disease and 69.0%; (95% CI 33.0–87.6) for severe RSV-related diseases, respectively. Pre-term birth occurred in 6.8% of the infants in the vaccine group and in 4.9% in the control group (relative risk, 1.37; 95% CI 1.08–1.74; *p* = 0.01); neonatal death occurred in 0.4% and 0.2, respectively (relative risk, 2.16; 95% CI, 0.62–7.56; *p* = 0.23) [20].

In accordance with Advisory Committee on Immunization Practices (2023), during the RSV season, pregnant women should receive the maternal RSVpreF vaccine between September and January. This vaccine is most effective for infants in the first few months of life. In particular, if a pregnant patient is not going to allow their newborn to be vaccinated with nirsevimab, the Centers for Disease Control and Prevention’s (CDC) Advisory Committee on Immunization Practices advised that they should receive an RSV immunization if they are between 32 and 36 weeks gestation and are expected to give birth during RSV season [21]. Pregnant individuals can receive the vaccination alongside other recommended vaccines without timing restrictions, including simultaneous vaccination at multiple anatomic sites on the same day. Finally, no information is available on the efficacy of the first lifetime dose in protecting infants born after subsequent pregnancies or the safety of extra doses given during subsequent pregnancies. More information is required to determine whether additional seasonal doses during subsequent pregnancies are recommended [21].

Of note, in light of the various potential strategies to prevent RSV-related illnesses (vaccination during pregnancy or the use of monoclonal antibodies), it is important that obstetricians discuss the relative benefits and possible disadvantages of both options with patients in order to consider the patient’s preferences regarding vaccination or eventual infant therapy. On the other hand, there is a lack of information in the literature comparing the effectiveness of monoclonal antibodies and the vaccination of the mother in preventing infant RSV-associated lower respiratory tract infections.

In rare cases, monoclonal antibodies such as nirsevimab or palivizumab may be administered to infants born to vaccinated mothers if the possible incremental benefit of administration is justified by the health care provider’s clinical judgment. These situations include infants born to mothers who may not have had an adequate immune response to vaccination or have conditions that reduce transplacental antibody transfer, infants who have lost maternal antibodies or extracorporeal membrane oxygenation, and infants at high risk for severe RSV disease [22,23].

Certain doubts remain about RSV vaccination during pregnancy. First of all, RSV-related disease primarily affects premature newborns with functional abnormalities of the lung epithelium; therefore, the preventive efficacy of vaccination during the third trimester of pregnancy will have no effect on neonatal outcome in this group. As a result, these high-risk infants will continue to require protection through other therapeutic approaches. Furthermore, the long-term effects of RSV immunization during pregnancy, as well as the extent to which it will lessen the global burden of RSV disease, remain unknown. Finally, the previously reported clinical trials did not examine the efficacy of vaccination in high-risk pregnancies, such as pregnancies that resulted in pre-term labor, multiple pregnancies, pregnancies complicated by autoimmune diseases or diabetes, or fetuses with severe congenital anomalies.

As a result, while promising progress has been made in the prevention of mortality and morbidity related to severe RSV diseases in infants by evaluating new therapeutic approaches based on monoclonal antibodies or vaccines, more research is required to evaluate the role of immunization during pregnancy and reduce the worldwide incidence of RSV-related lower respiratory tract diseases. This research will also produce data that can be used in everyday clinical settings and increase the acceptability of vaccines during pregnancy.

## Data Availability

Data sharing is not applicable to this article as no datasets were generated or analyzed during the current study.
